# The Anti-Viral and Anti-Inflammatory Properties of Edible Bird’s Nest in Influenza and Coronavirus Infections: From Pre-Clinical to Potential Clinical Application

**DOI:** 10.3389/fphar.2021.633292

**Published:** 2021-05-07

**Authors:** Kien Hui Chua, Isa Naina Mohamed, Mohd Heikal Mohd Yunus, Norefrina Shafinaz Md Nor, Khidhir Kamil, Azizah Ugusman, Jaya Kumar

**Affiliations:** ^1^ Department of Physiology, Faculty of Medicine, Universiti Kebangsaan Malaysia, Cheras, Malaysia; ^2^ Department of Pharmacology, Faculty of Medicine, Universiti Kebangsaan Malaysia, Cheras, Malaysia; ^3^ Department of Biological Sciences and Biotechnology, Faculty of Science and Technology, Universiti Kebangsaan Malaysia, Bangi, Malaysia

**Keywords:** edible bird’s nest, anti-viral, anti-infammatory, influenza, coronavirus, COVID-19, cytokine, bird nest

## Abstract

Edible bird’s nest (BN) is a Chinese traditional medicine with innumerable health benefits, including anti-viral, anti-inflammatory, neuroprotective, and immunomodulatory effects. A small number of studies have reported the anti-viral effects of EBN against influenza infections using *in vitro* and *in vivo* models, highlighting the importance of sialic acid and thymol derivatives in their therapeutic effects. At present, studies have reported that EBN suppresses the replicated virus from exiting the host cells, reduces the viral replication, endosomal trafficking of the virus, intracellular viral autophagy process, secretion of pro-inflammatory cytokines, reorient the actin cytoskeleton of the infected cells, and increase the lysosomal degradation of viral materials. In other models of disease, EBN attenuates oxidative stress-induced cellular apoptosis, enhances proliferation and activation of B-cells and their antibody secretion. Given the sum of its therapeutic actions, EBN appears to be a candidate that is worth further exploring for its protective effects against diseases transmitted through air droplets. At present, anti-viral drugs are employed as the first-line defense against respiratory viral infections, unless vaccines are available for the specific pathogens. In patients with severe symptoms due to exacerbated cytokine secretion, anti-inflammatory agents are applied. Treatment efficacy varies across the patients, and in times of a pandemic like COVID-19, many of the drugs are still at the experimental stage. In this review, we present a comprehensive overview of anti-viral and anti-inflammatory effects of EBN, chemical constituents from various EBN preparation techniques, and drugs currently used to treat influenza and novel coronavirus infections. We also aim to review the pathogenesis of influenza A and coronavirus, and the potential of EBN in their clinical application. We also describe the current literature in human consumption of EBN, known allergenic or contaminant presence, and the focus of future direction on how these can be addressed to further improve EBN for potential clinical application.

## Introduction

Edible bird’s nest (EBN) is a nest produced from the salivary secretion of swiftlets such as *Aerodramus sp.* and *Callocalia sp.*, which are commonly found in the South-East Asian regions. To date, a myriad of studies reported various therapeutic potentials of EBN, including anti-aging (Hwang et al., 2020), anti-inflammatory (Vimala et al., 2012), anti-viral (Guo et al., 2006), immunomodulatory (Haghani et al., 2016), anti-oxidant (Yew et al., 2014), and so forth. Given its broad health benefits, EBN is a highly sought-after medicinal food in Asia (Abidin et al., 2011; Looi and Omar, 2016).

Recent reports on global mortality caused by seasonal influenza indicate up to 290,000 to 650,000 deaths associated with respiratory illnesses alone (Iuliano et al., 2018), and the Global Burden of Disease Study (GBD) attributed 99,000 to 200,000 annual deaths from lower respiratory tract infections directly to influenza (GBD 2017 Influenza Collaborators, 2019). As of October 2020, a novel coronavirus, otherwise known as severe acute respiratory syndrome coronavirus 2 (SARS-CoV-2), has infected more than 50 million and killed no less than one million worldwide (Worldometer, 2020).

Through antigenic shift and drift, influenza viruses learn to evade inherent immune responses (Kim et al., 2018), hence continuous reformulation of immunization and vaccine persists (Principi et al., 2019). Moreover, suboptimal vaccine-induced immune responses in vulnerable populations like the elderly and young children also present a major strain on the management of influenza infections (Sano et al., 2017). The primary drug of choice for influenza, anti-viral medications are dogged by treatment-resistant viral strains (Hayden et al., 2018; Omoto et al., 2018), expensive treatment cost (Yang, 2019), and adverse effects (Witcher et al., 2019), despite their proven benefits. Whereas for SARS-CoV-2, at present, the efficacy of vaccines or anti-viral medications is plagued by fast mutating nature of the virus.

Over the past decades, there has been a growing interest in the use of natural products for their medicinal values (Kamil et al., 2018; Hamid et al., 2020; Kamil et al., 2020; Prom-In et al., 2020). EBN, is an emerging food product with known anti-viral effects, especially against Influenza A virus (IAV) (Guo et al., 2006; Haghani et al., 2016; Haghani et al., 2017), the most common cause of the global pandemic. EBN significantly reduced viral titer, viral-induced hemagglutination (Haghani et al., 2017), virus-binding activity (Guo et al., 2006), and virus-induced endocytosis and autophagosomes (Haghani et al., 2017). In addition to anti-viral, EBN also demonstrated anti-inflammatory (Vimala et al., 2012), anti-oxidant (Yew et al., 2014), and immunomodulatory properties (Haghani et al., 2016), indicating its potential as an all-around prophylactic or therapeutic option for influenza, SARS-CoV-2 and its associated complications, such as cytokine storm. In this review, we described the pathogenesis of IAV and SARS-CoV-2 infections, current anti-viral medications in clinical practice for both infections, chemical compositions, and potential benefits of EBN as an anti-viral, anti-inflammatory, anti-oxidant, and immunomodulatory agent in the treatment of IAV and SARS-CoV-2 infections.

## Influenza A

The IAV is an enveloped virus with segmented negative sense, single-stranded RNA materials. The primary glycoprotein antigen on the virion surface, hemagglutinin (HA), and neuraminidase (NA) are the target of inherent immune response and many anti-viral medications (McAuley et al., 2019). To date, 18 types of H antigen, and 11 N antigens have been identified (Tong et al., 2013). Throughout history, IAV is known to cause various pandemics, such as the H1N1 virus pandemic in 1918 (Saunders-Hastings and Krewski, 2016), H2N2 in 1957, H3N2 in 1968, and another H1N1 virus pandemic in 2009 (Guan et al., 2010).

IAV, through HA on the surface of the virion, binds to the terminal sialic acid residues on mucins (Ma et al., 2017), which is released by the actions of NA cleaving off the terminal sialic acid residues (Yang et al., 2014), leading to endocytosis of IAV into the host cell past the mucosal layer. Once in, HA undergoes conformational changes to expose fusion peptide to promote viral endosomal membrane fusion, and IAV core undergoes acidification *via* protein entry through the M2 ion channels, allowing vRNPs to be released into the cytoplasm (Padilla-Quirarte et al., 2019). The IAV genome is transcribed and translated to synthesize HA, NA, M2 ion channel, matrix protein (M1), nuclear export protein (NEP), polymerases (PB1, PB2, PA), nucleoprotein (NP), PB1-F2, PA-X, and non-structural protein 1 (NS1). The synthesized viral particles attach to the host cell membrane due to the interaction between HA and sialic acids and released by the catalytic actions of NA on terminal sialic acid residues (Krammer, 2019).

The major types of sialic acid present in the terminal side of the glycans of mammalian and avian glycoproteins and glycolipids are N-acetylneuraminic acid (Neu5Ac; mostly humans) and N-glycolylneuraminic acid (Neu5Gc) (For review Long et al., 2019). HA from human-adapted viruses is known to bind to α2-6-linked sialic acid, whereas HA from avian influenza viruses binds to α2-3-linked sialic acid (Rogers and Paulson, 1983). The X-ray crystallographic and glycan microarray binding studies revealed a receptor binding site of HA from human-adapted viruses contain a bulkier cis conformation adopted by α2-6-linked sialic acid, compared to the HA of avian influenza viruses with thin and straight trans conformation by the α2-3-linked sialic acid (Shi et al., 2014; Lipsitch et al., 2016). Studies also have reported both α2-3 and α2-6 sialic acid linkages in the human lung and bronchus (Walther et al., 2013), α2-6 linkages in the respiratory tracts of ferrets and pigs (Nelli et al., 2010; Jia et al., 2014), and higher expression of α2-3 sialic acid linkages in non-human primates and mice (Gagneux et al., 2003; Ning et al., 2009). Other features of glycans also determine the interaction between virus and host, such as the presence of other sugar moieties or functional groups, length of sialic acid presenting glycans (Long et al., 2019), and second binding site in addition to a usual catalytic sialic acid binding site of NA, such as the hemadsorption (Hd) site (Uhlendorff et al., 2009). More recent findings suggest the binding to the secondary site may occur prior to the binding to the primary site where the enzymatic cleavage occurs (Durrant et al., 2020).

## Anti-Viral Medications Against IAV

Vaccination is the primary mode of prevention against influenza. Though, most of the vaccines are not 100% effective as the influenza viruses are constantly evolving (Hurt, 2014). Hence, anti-viral medications are in continuous development given their importance in the management of influenza infections, particularly during the initial phases of a pandemic when vaccines are still in the making. Table 1 shows a comprehensive overview of various anti-virals used to treat IAV infection.

**TABLE 1:** Anti-Viral medications for the treatment of Influenza A virus.

Chemical name	Trade name	Mechanism of action	Infectious agent	Resistance reported	Side effects	Efficacy
Baloxavir marboxil	Xofluza	Selective inhibition of cap-dependent endonuclease activity of the influenza virus polymerase acidic (PA) protein Heo (2018); Takashita et al. (2019); Noshi et al. (2018); Omoto et al. (2018)	Influenza A, B	I38X amino acid substitutions in the influenza PA protein Hayden et al. (2018); Gubareva et al. (2019); Takashita et al. (2019).	The most common; diarrhea. Other reported; bronchitis, nausea, sinusitis, increase in AST and ALT, and headache Hayden et al. (2018); Ison et al. (2020); Shionogi and Co. Ltd (2018).	Oral capsule.
						Influenza A or B in otherwise healthy adults and children (aged ≥12 years).
						Influenza A or B in adults and children (aged ≥12 years) with high risk of influenza complications. Hayden et al. (2018); Ison et al. (2020).
Oseltamivir	Tamiflu	Specific inhibitors of enzyme neuraminidase, which promotes virus spread in the respiratory tract and virus release from the infected cells Gubareva et al. (2000); Ohuchi et al. (2006); Hama (2016).	Influenza A, B	i) Mutations within or close to the HA receptor-binding site gene reduces successful virus budding to cellular receptors, which reduces dependence on NA function Gubareva et al. (2000).	Diarrhea, bronchitis, nausea and sinusitis Hayden et al. (2018); Ison et al. (2020); Gubareva et al. (2000); Hama (2016), insomnia, vertigo Moscona (2005); Hama (2016).	Oral capsule and suspension,
				Ii) Substitutions of amino acid at the conserved residues in the active site of NA enzyme -mutation H274Y, R292K and N294S Gubareva et al. (2000); Wang et al. (2002).		Influenza A or B in uncomplicated acute illness patients aged >1 year Ward et al. (2005); Samson et al. (2013).
Zanamivir	Relenza	Specific inhibitors enzyme neuraminidase. Gubareva et al. (2000); Ohuchi et al. (2006); Hama (2016).	Influenza A, B	D151 A/E/G/V mutations in inf A viruses Sheu et al. (2008).	Headache, dizziness nausea, diarrhea vomiting sinusitis, bronchitis, cough Moscona (2005); Samson et al. (2013).	Oral inhalation. Influenza A or B treatment, approved for patients older than 7 years Samson et al. (2013).
				E119G/D/A substitutions in influenza B viruses Jackson et al. (2005).		
Peramivir	Rapivab	Specific inhibitors enzyme neuraminidase. Gubareva et al. (2000); Ohuchi et al. (2006); Hama (2016).	Influenza A, B	H274Y mutation in influenza A viruses, Renaud et al. (2010); Scott (2018).	Diarrhea (mild to moderate), nausea, and vomiting, cough, and pyrexia Komeda et al. (2015); Scott (2018).	Parenteral. Influenza A or B treatment, approved for patients of all ages Samson et al. (2013).
				V94I and R152K: mutation in influenza B viruses, from a pediatric patient Yoshida et al. (2011).		Uncomplicated influenza in children from the age of 2 years and adults Scott (2018).
Favipravir	Avigan	Inhibits RNA polymerase activity by preventing RNA elongation Furuta et al. (2013)	Influenza A, B	V43I in PB1 in influenza A viruses Cheung et al. (2014).		
				K229R in PB1 subunit of influenza A virus Goldhill et al. (2018).		

The anti-viral baloxavir marboxil (XofluzaTM) is a cap-dependent endonuclease inhibitor that was developed to treat IAV or B infection (Shionogi and Co. Ltd, 2018). Following Japan in 2018, baloxavir is approved in many other countries, including the United States in 2019 (Takashita et al., 2019). The mechanism of action of baloxavir is dissimilar to neuraminidase inhibitors, where it suppresses the proliferation of virus through inhibition of mRNA synthesis initiation (Koszalka et al., 2017; Noshi et al., 2018). Administration of a single dose of baloxavir within the 48 h of symptom onset rapidly improved the symptoms, with fewer influenza-related complications. Moreover, a single dose of baloxavir also reduced viral replication in high-risk adult and adolescent outpatients with uncomplicated influenza (Portsmouth et al., 2017; Ison et al., 2020). Children older than 12 years of age and adults suffering from IAV or B generally well-tolerated a single dose of baloxavir (Heo, 2018). Otherwise, the most commonly associated adverse effects with baloxavir are diarrhea, nausea, bronchitis, and sinusitis (incidence of ≤3%) (Shirley, 2020), and increase in AST, ALT, and headache (incidence <1%) (Shionogi and Co. Ltd, 2018).

Oseltamivir (TamifluTM) is a specific inhibitor of IAV that has proven clinically effective in adults and children (as young as 1 year) for the chemoprophylaxis and treatment of IAV and B infections (Ward et al., 2005). Oseltamivir is available in oral form (75 mg twice daily used by a large population of patients) (McNicholl and McNicholl, 2001) and intravenously for those unable to tolerate oral dosing (Kamali and Holodniy, 2013). Oral administration of Oseltamivir (75 mg twice daily) significantly reduced the severity and duration of symptoms (McNicholl and McNicholl, 2001). Oseltamivir is relatively well tolerated, with the most commonly associated side effects such as abdominal pain, vomiting, and transient nausea in 5–10 percent of patients studied (Moscona, 2005). Others have also reported Oseltamivir-related serious adverse effects, such as sudden deaths and accidental deaths due to abnormal behaviors (Hama, 2016). Resistance to the drug has also been reported in the past, among patients who have been exposed to the drug previously, and also without any prior exposure (Kamali and Holodniy, 2013).

Zanamivir (Relenza™) is the first neuraminidase inhibitor to be developed, with a high affinity for the neuraminidase binding site (McKimm-Breschkin, 2013). Zanamivir has poor oral bioavailability, hence administered with an inhaler device (Diskhaler) (Diggory et al., 2001). Therefore, the inspiration flow determines the amount of drugs reaching the respiratory tract, which is a limitation, especially for intubated patients (Ison, 2010). For patients older than seven years of age, Zanamivir (10 mg) is inhaled twice daily for 5 days, whereas prophylaxis is given to patients older than 5 years of age once daily for 10–28 days (Samson et al., 2013). Zanamivir reduces the period of symptom alleviation by 10–24% (0.5–1.25 days) (McNicholl and McNicholl, 2001).

Peramivir (Rapivab®) is a novel cyclopentane neuraminidase inhibitor with potent and selective inhibitory action against IAV and B virus’ NA, with similar or more potent *in vitro* inhibitory effects against IAV and B, than zanamivir or oseltamivir (Fage et al., 2017). In 2014, the Food and Drug Administration approved the use of peramivir for the treatment of acute uncomplicated influenza in patients 18 years and older (Alame et al., 2016). Due to its poor oral bioavailability, peramivir is offered only as an intravenous formulation, and its inhibitory activity against influenza is slightly lower than oseltamivir and zanamivir (Fage et al., 2017). Pediatric and adult patients with uncomplicated influenza generally well tolerate a single dose of peramivir. The common adverse reactions reported are nausea (2.4% of patients) and reduced neutrophil counts (3.2%) (BioCryst, 2018). In children (frequency 1 to <10%), the most frequently associated adverse reactions are rashes at injection sites, tympanic membrane hyperemia, pyrexia, pruritus, and psychomotor hyperactivity (BioCryst, 2018).

## Severe Acute Respiratory Syndrome-Related Coronavirus

Coronavirus Disease 2019 (COVID-19) caused by the severe acute respiratory syndrome coronavirus (SARS-CoV-2) has infected more than 50 million, and killed at least 1.2 million worldwide, as of November 2020 (Worldometer, 2020). The Timeline Outbreak by SARs-COV-2 viruses began when severe cases of pneumonia were reported in Wuhan City of Hubei Province in China. Investigations into etiological agents led to the Hunan seafood wet market in Wuhan, China where the sample tested from this wet market was found to be positive for SARS-CoV-2, which was initially known as 2019-nCoV before being renamed as SARS-CoV-2 (Ralph et al., 2020; Wu et al., 2020).

Coronavirus is made of the Membrane (M), Envelope (E), Nucleocapsid (N), and spike protein (S), and two large polyproteins (Yoshimoto, 2020). Among them, the ones that enabled the coronavirus to infect and replicate in humans are S and N proteins. The S protein of SARS-CoV-2 has a receptor-binding domain (RBD) similar to SARS-CoV, which binds to the same receptor Angiotensin-converting Enzyme 2 (ACE2) in humans, but with 10-20-fold higher affinity (Wrapp et al., 2020; Zhang and Holmes, 2020), though MERS-CoV expresses a different receptor, dipeptidyl peptidase 4 or DPP4 (Wang et al., 2013). The binding of the S protein to ACE2 enables the entry of SARS-CoVs into the human cell (Letko et al., 2020), whereas N protein is vital for replication and assembly (V’kovski et al., 2020).

## Anti-Viral Agents Against SARS-CoV-2

Remdesivir (RDV) (sold under the trade name Veklury) gained prominence as it is the first anti-viral drug to be approved by the US FDA to be used as a treatment option for SARS-CoV-2. Initially, RDV was developed as a general anti-viral drug for hepatitis C and Ebola, but results were not encouraging. RDV acts by inhibiting the coronavirus’s RNA synthesis through delayed chain termination. RDV was touted as a potential anti-viral therapy option for SARS-CoV-2 early in the pandemic. Trials were quickly initiated. Intravenous RDV was found to have been numerically better but not statistically significant in mortality, clinical improvement, and time taken to clear off the virus (Wang X. et al., 2020). Grein et al. in another trial reported that 68% (36 of 53 patients) of patients showed improvement with regards to oxygen support on day 18, and 84% had significant clinical improvement by day 28 in severe Covid-19 cases (Grein et al., 2020). In both trials, adverse effects were mostly well tolerated with common adverse events reported as constipation, increased total bilirubin, anemia, diarrhea, rash, and hepatic enzymes. Severe adverse events include septic shock, acute kidney injury, and multiple organ dysfunction.

The anti-viral favipiravir (FPV) (sold under the trade name Avigan and Fabiflu) is developed by Toyama Chemical (Japan), has been sold in Japan for the treatment of influenza since 2014. It was one of the early candidates recognized for the pharmacotherapy of SARS-CoV-2 and was used in Wuhan as a treatment option. Since then, a few trials have been initiated in various countries with varying degrees of success. FPV competitively inhibits RNA-dependent RNA polymerase in the virus (Furuta et al., 2013). FPV profoundly improved the latency to relief for cough and pyrexia in patients with COVID-19 (Chen C. et al., 2020). FPV also improved clinical recovery in Day 7 in moderately severe COVID-19 patients as well as decreased auxiliary oxygen therapy and decreased the incidence of dyspnea (Chen L. et al., 2020). Another trial by Cai et al. (2020) reported a significant decrease in the time SARS-CoV-2 viral clearance and improvement in chest imaging in comparison to ritonavir or lopinavir (Cai et al., 2020). FPV is a well-tolerated drug with the most commonly reported adverse event of elevated serum level of uric acid and had a better safety profile compared to lopinavir or ritonavir (Cai et al., 2020; Chen C. et al., 2020).

Ritonavir (LPV/r) and Lopinavir is a fixed-dose anti-viral combination used for the pharmacotherapy and prevention of HIV/AIDS. LPV/r acts by inhibiting the protease activity of viruses. LPV/r was also touted early in the epidemic as an anti-viral treatment for SARS-CoV-2. A preclinical *in vitro* study by Kang et al. reported lower viral load in LPV/r treated group and concluded profound inhibition of SARS-CoV-2 at plasma concentration (Kang et al., 2020). Many trials have since concluded on its efficacy. Wang et al. reported significant alleviation of pneumonia-associated symptoms (Wang Y. et al., 2020). Cao et al. reported overall lower 28-days mortality (19 vs 25%) on patients receiving LPV/r but it was not statistically significant (Cao et al., 2020). Similarly, Chen C. et al., 2020, in a retrospective study reported no profound difference between LPV/r treated and control groups in symptom improvement or reduction in viral loads (Chen L. et al., 2020). Most studies reported that LPV/r anti-viral is a well-tolerated drug with gastrointestinal adverse effects as the most commonly reported adverse effects. Table 2 shows a comprehensive overview of various anti-viral medications for SARS-CoV-2.

**TABLE 2:** Antiviral medications for the treatment of SARS-CoV-2.

Chemical name	Trade name	Mechanism of action	Infectious agent	Resistance reported	Adverse effects	Efficacy
Rem-desivir	Veklury	Inhibits the RNA synthesis by virus through delayed chain termination for all three coronaviruses’ RdRp.	SARS-CoV-2	In a review, the authors concluded that there is a high genetic barrier for remdesivir to develop resistance as well as decreased fitness and pathogenicity in remdesivir-resistant mutants, which further encourage the therapeutic potential of remdesivir in the treatment of newly emerging COVID-19 Ko et al. (2020).	The most commonly reported were hypoalbuminemia, anemia, thrombocytopenia, constipation, hypokalemia, and elevated total bilirubin Wang X. et al. (2020).	Intravenous RDV had a numerically better improvement, but did not profoundly reduce the time taken for clinical improvement, mortality, or time for virus clearance in serious COVID-19 cases in comparison to placebo Wang Y. et al. (2020).
					Increase in hepatic enzymes, rash, renal impairment, hypotension, diarrhea. *Generally, AE were more commonly associated in patients receiving invasive ventilation Grein et al. (2020).	Over a median follow-up of 18 days after the first dose of RDV, 36 of 53 patients (68%) showed an improvement in the category of oxygen support, whereas 8 of 53 patients (15%) worsened Grein et al. (2020).
					The most common SAE were septic shock, acute kidney injury, hypotension, and multiple-organ-dysfunction syndrome Grein et al. (2020).	By 28 days of follow-up (RDV group), there were 84% cumulative incidence of clinical improvements in severe cases of Covid-19 Grein et al. (2020).
Favi-piravir	Avigan	Competitively inhibits the RNA-dependent RNA polymerase Furuta et al. (2013).	SARS-CoV-2	None	The most frequently observed FPV-associated AE was raised serum uric acid Chen C. et al. (2020).	Favipiravir profoundly attenuated the latency to relief for pyrexia and cough in COVID-19 patients.
						Based on post-hoc analysis, Favipiravir effectively improved clinical recovery rate at Day 7 in moderate COVID-19 patients compared to Arbidol. Similar effect was diminished in severe/critical COVID-19 cases.
					The rate of adverse events in patients receiving favipiravir was significantly lower (11.4% vs. control 55.6%; *p* < 0.01) Khamis et al. (2021).	Based on a post-hoc analysis, in moderate COVID-19 patients, Favipiravir was associated with:
						-Reduced noninvasive mechanical ventilation rate or auxiliary oxygen therapy with marginal significance (*p* = 0.0541).
						- Profoundly reduced *de novo* incidences of dyspnea Chen L. et al. (2020).
						In China, an open-label non-randomized trial of COVID-19 80 patients reported a profound decrease in the time to SARS-CoV-2 viral clearance in patients treated with favipiravir compared with historical controls treated with lopinavir/ritonavir
						The FPV arm also showed profound improvement in chest imaging compared with the control arm, with an improvement rate of 91.43 vs. 62.22% (*p* = 0.004) Khamis et al. (2021).
Lopinavir	Kaletra	Inhibits the protease activity of coronavirus.	SARS-CoV-2	None	Gastrointestinal adverse events were more common in the lopinavir–ritonavir group Cao et al. (2020).	One study showed the positive effects of LPV/r therapy:
	-Fixed-dose combination with ritonavir (LPV/r)					
	- Ritonavir helps to stabilize lopinavir					
						Four COVID-19 patients were given antiviral treatment including LPV/r. Following treatment, three of them showed marked improvement in pneumonia-associated symptoms. Two patients were confirmed to be COVID-19 negative and discharged, and one of whom was negative for the virus at the first test Wang Z. et al. (2020).
						A combination treatment with adjuvant drugs and LPV/r has better therapeutic effects in reducing the body temperature and restoring normal physiological mechanisms with no evident toxic and side effects Ye XT. et al. (2020).
						The efficacy of lopinavir/ritonavir treatment was reported from a single case report from the index patient treated in Korea, whose viral titers diminished following treatment Lim et al. (2020).
	*Combination of (LPV/r) has been proposed for the treatment of coronaviruses because of its potential effect on viral replication at the cellular level.					An *in vitro* study, using Vero cells:
						The severity of cytopathic effects was lesser in lopinavir/ritonavir-treated cells, and viral load was profoundly decreased in this group compared to the control (*p* < 0.001).
						Lopinavir/ritonavir significantly inhibited SARS-CoV-2 *in vitro* at its usual plasma concentration Kang et al. (2020).
						Lopinavir–ritonavir co-administration did not profoundly accelerate clinical improvement, decrease mortality, or reduce throat viral RNA detectability in serious Covid-19 cases.
						Lopinavir-ritonavir treatment recorded lower 28-days mortality (19 vs. 25%), however without any significant between-group differences Cao et al. (2020).
						A retrospective study enrolling 134 novel coronavirus pneumonia (NCP) patients reported no significant difference in improvement of symptoms or reduction in viral loads between control (*n* = 48), LPV/r-treated group (*n* = 52), and Abidol-treated group (*n* = 34) Chen L. et al. (2020).
						*(article in Chinese language)
Ritonavir	Norvir	Inhibit protease	SARS-CoV-2	None.	Same as the lopinavir	Same as the lopinavir
		Inhibits the metabolizing enzyme CYP450 3A and therefore increases the half-life of lopinavir (other antiretroviral drugs which it is co-administered with) Chandwani and Shuter (2008).		Resistance reported for HIV:		
				The valine at position 82 (Val 82) in the active site of the human immunodeficiency virus (HIV) protease mutates in response to therapy with the protease inhibitor ritonavir.		

## Anti-Viral Effects of EBN

The anti-viral properties of EBN were tested using *in vitro* and *in vivo* models and compared across the efficacy of standard anti-viral medications such as oseltamivir and amantadine (Haghani et al., 2017) (Table 3). In anti-viral cell culture studies, EBN did not produce any cytotoxic effects up to 50 mg/ml (Nuradji et al., 2018), whereas some reported CC50 of EBN extracts at a lower concentration range 27.2–32 mg/ml (Haghani et al., 2017). For anti-viral *in vivo* studies, concentration in the range of 5–2000 mg/kg was tested in 8–10 weeks old female mice *via* oral gavage and was shown to be free of toxicity and mortality at the end of 14 days of the treatment period (Haghani et al., 2016). The EBN concentration range of 12.5 mg/ml (Haghani et al., 2017), 0.5–4,000 μg/ml (Guo et al., 2006), and 15–35 mg/ml (Nuradji et al., 2018) was employed in most anti-viral investigations against IAV strains of H1N1, H3N2, and H5N1, respectively.

**TABLE 3:** Antiviral effects of EBN.

References	Cell Lines and Virus strain	Origin of EBN and dosage	Experimental design	Analysis	Summary of findings
Guo et al. (2006)	A/Shizuoka/450/05 (H3N2) and A/Shizuoka/451/05 (H3N2) strains.	Natural cave EBN.	Sample 1 (S1) was collected from a bird’s nest from a natural cave.	Hemagglutination inhibition (HAI) assay.	EBN extracts treated with pancreatic enzyme (both S1 and S2) had significant HAI activities. S1 EBN extract inhibited strain A/Aichi/2/68 stronger than S2 and treatment with neuraminidase reduced their effects.
	Madin-Darby canine kidney (MDCK) cells and Human lung carcinoma	House-cultured EBN.	Sample 2 (S2) was collected from house-cultured bird’s nest.	Sialidase inhibition assay.	Virus binding activity of S1 EBN was greater than S2, and treatment with neuraminidase markedly reduced binding activity of EBN extract S1.
	A549 cells.			Neutralization assay through lactate dehydrogenase (LDH) level.	EBN extract had no effects on the neuraminidase activity of influenza viruses, and remained side-effects free in all experiments.
				Western blot (detected the binding activity of the viruses to the glycoprotein from the EBN extracts against anti-H3N2).	Human influenza virus strain A/Aichi/2/68 (H3N2) bound to sialyl 2–3 and 2–6 galactose linkages in salylglycoproteins and sialylglycolipids.
				Fluorometric HPLC analysis for sialic acid and lectin blotting method to determine sialic acid linkages.	Neu5Ac2-3Gal linkage was the major molecular species of sialic acid in the EBN extract.
Haghani et al., (2016)	*In vitro*	House nest from teluk Intan,	1) House nest EBN with no enzymatic treatment.	*In vitro*	*In vitro*
	Influenza A virus, strain A/PR/8/34 (H1N1).	Perak, Malaysia.	2) House nest EBN with Pancreatin F-treatment.	RNA changes of viral genes (NS1, NA, NP, and M2)	Antiviral effects:
	Madin Darby canine kidney (MDCK) cell line (viral propagation).	Cave nest from Gua Madai caves, Lahad Datu, Sabah, Malaysia.	3) Cave nest EBN with no enzymatic treatment.	Cytokines (TNFα, IL6, IFNβ, IL27, and CCL2)	EBN was found to reduce the copy number of intracellular NA gene and extracellular NS1 gene. However, EBN increased M2 gene and had no effect on NP gene.
	*In vivo*	*In vivo*	4) Cave nest EBN with neuraminidase-treatment.	NFκB activation protein (NFAP)	Immunomodulatory effects:
	A/Peurto Rico/8/1934 (H1N1).	100 mg/kg (1 ml/100g body weight using oral gavage).	*In vitro*	*In vivo*	EBN can regulate the excessive innate inflammatory reaction by significantly reducing CCL2, IL-6, and IFN-β expression while significantly increasing the IL-27 and TNF-α expression.
			Infected MDCK cell lines were treated with EBNs and commercial antiviral drugs (Oseltamivir phosphate and Amantadine hydrochloride) for 48 h.	Viral load in lungs	EBN profoundly increased NFκB activation protein (NFAP).
			*In vivo*	Blood cytokine levels (TNFα, IFNγ, IL1β, IL2, IL4, IL6, IL10, IL12 (p70), and IL15).	*In vivo*
			Mice were challenged with intranasal 10^5^ TCID50 of A/Peurto Rico/8/1934 (H1N1). The mice were treated with EBN or oseltamivir phosphate (OSE) 4 h before viral challenge.		Pretreatment of EBN and treatment of OSE both reduced NA copy number in lung.
					EBN significantly decrease IFN-γ and increased IL-4, IL6, IL-12, and TNF-α by day 3.
					EBN reduced IL-10, and IL-12 by day 7.
Haghani et al. (2017)	Infleunza A virus strain A/Puerto Rico/8/1934 (H1N1).	House nest EBN from teluk Intan, Perak, Malaysia.	1) House nest EBN with no enzymatic treatment.	MTT assay for EBN cytotoxicity.	EBN CC50 ranged from 27.5 to 32 mg/ml, while IC50 ranged 2.5–4.9 mg/ml, amantadine: CC50: 4,523.4 mg/ml, oseltamivir: CC50: 14,373.2 mg/ml.
	Madin Darby Canine Kidney (MDCK) cell line.	Cave nest EBN from Gua Madai caves, Lahad Datu, Sabah, Malaysia.	2) House nest EBN with Pancreatin F-treatment.	Hemagglutination assay.	EVB decreased the influenza virus hemagglutination activity in a dose dependent manner.
			3) Cave nest EBN with no enzymatic treatment.	Endosomal trafficking through immunoblotting of Rab5, RhoA and LC3 translocation and immunofluorescent of actin cytoskeleton and lysosomal bodies.	EBNs could significantly (*p* < 0.05) decreased the expression of Rab5 protein, in par with oseltamivir. Amantadine had no effect on Rab5 protein in infected and normal cells.
			4) Cave nest EBN with neuraminidase-treatment.		EBNs markedly (*p* < 0.05) reduced the RhoA as similar to Amantadine and oseltamivir. EBNs failed to reduce LC3 protein.
			Infected MDCK cell lines were treated with EBNs to measure EBN cytotoxicity and its effects on autophagy process and endosomal trafficking of influenza virus.		EBNs normalized the cellular shapes and reoriented the actin filaments, and reduced densities of actin filaments. At the same time, EBN increased the density and the number of lysosomes in both infected and non-infected cells.
Nuradji et al. (2018)	H5N1 HPAI virus clade 3.2.3 A/DK/BNY/F.2014P1 in chicken embryonic eggs.	Thirteen samples of EBN extract from 12 swiftlet houses from Indonesia were used.	Thirteen samples of EBN extract were used.	Cytotoxic effects of EBN extract:	Up to 50 mg/ml of EBN did not generate cytotoxicity in cell culture. CC50 of EBN extract was detected at more than 50 mg/ml. EBN concentrations begin to show HI activity at 12 μg/ml.
	Vero cell (African Green Monkey Kidney Cells).		Monolayer Vero cells was exposed with different concentrations of EBN extract for 96 h.	Cell shrinkage, blebbing membranes, ballooning cell, chromatin condensation, and cytoplasmic vacuolation.	EBN with low concentration (25 mg/ml) showed a high HI titer compared with EBN of higher concentration (35 mg/ml).
				Hemagglutination-inhibition test.	
				Viral titer.	

Most of the *in vitro* anti-viral studies of EBN used the water extraction method to prepare the EBN test sample. Usually, EBN is dried at a temperature higher than ambient for few hours to one day before grinding and filtering EBN with wire mesh to separate the feather and impurities. The ground EBN was then soaked in water or water-based buffer before heated to release the water-soluble parts of the EBN. Pancreatin enzyme was commonly used to simulate the gastrointestinal digestion process to break down the larger protein components of EBN. For *in vivo* model, EBN extract without the pancreatin enzyme is preferred in the experiment since the animal gut could do the job (Haghani et al., 2016). As the anti-viral effect of the EBN depends on the amount, concentration, and bioavailability of the functional groups; the location of EBN harvest, seasoning effect, and extraction methods can affect the study findings. Table 4 shows an overview of the analysis of EBN composition in various studies.

**TABLE 4:** Analysis of EBN composition.

References	Source of EBN	Preparation of EBN	Methods of analysis	Results
Guo et al. (2006)	Indonesia,	**Grinding**	Fluorometric determination of 5-N-acetyl-neuraminic acid (Neu5Ac) and 5-N-glycolylneuraminic acid (Neu5Gc) through high-performance liquid chromatography (HPLC) method.	S1 has Neu5Ac as the major molecular species of sialic acid.
	Sample 1 (S1)- natural cave	Nests were dried at 70°C for 16 h, ground and sifted to remove the foreign substances and feathers.		S2 has O-acetyl sialic acid species.
	Sample 2 (S2)–house -cultured	**Heating**		The sialyl-glycoconjugates of S1 have Neu5Ac2-3Gal linkages and S2 have an O-acetylated Neu5Ac.
		Nests were placed in 5°C distilled water for 16 h and heated at 60°C for 30–60 min.		The sialyl glycoconjugates with Neu5Ac2-3Gal linkages could inhibit influenza virus infection.
		**Enzymatic treatment**		
		1) Samples were exposed to pancreatin F (final concentration of 0.5 mg/ml) at 45°C for 4 h at pH 8.5–9.0, and then heated at 90°C for 5 min for enzyme inactivation.		
		2) Samples were exposed to neuraminidase (final concentration of 0.5 mg/ml) at 45°C for 2 h at pH 8.5–9.0 and then heated at 90°C for 5 min for enzyme inactivation.		
		**Filtration and Storage**		
		The extracts were filtered and freeze-dried at -80 °C after 48 h, and then stored at -80°C for further usage.		
Yagi et al. (2008)	Indonesia	The EBN extract were prepared based on Guo et al. (2006).	HPLC mapping method, MALDI-MS/MS sequencing, gas chromatography– electron impact methylation analysis.	The findings of tri-antennary N-glycan as a major component suggested that the sialylated N-glycans of EBN may contribute to the antiviral effects of EBN.
Halimi et al. (2014)	EBN from Pahang and terengganu by Nest Excel	**Storage**	Nutritional composition of moisture, protein, carbohydrate, ash and fat content.	Protein was the major components in EBN samples (58.55%, Pahang; 55.48%, terengganu) followed by carbohydrate, moisture, ash and fat.
		EBN samples were stored in air-tight container at room temperature.	Amino acid composition	Glutamic acid (9.61%), aspartic acid (6.34%), lysine (5.44%) and leucine (5.30%) were the major amino acids found in the EBN samples.
			Total Solubility	The total solubility of EBN increased with boiling time; 4 h gave the most soluble form.
Hou et al. (2015a)	Sabah and Sarawak, Malaysia	**Grinding**	ELISA for lactoferrin (LF) and ovotransferrin (OVF).	The concentrations of LF and OVF -House white EBN (Sabah):
		Nests were dried in an oven at 50 °C for three days and finely ground.		LF: 4.68 ± 0.57 μg/mg, OVF: 10.23 ± 0.72 μg/mg.
		**Ultrasonication and Centrifugation**		Cave white EBN (Sabah):
		Samples were dissolved into 1000 ml of dd H2O, and sonicated in an ice followed by centrifugation.		LF: 4.27 ± 0.49 μg/m, OVF: 10.63 ± 0.90 μg/mg.
		**Filtration and Storage**		Cave black EBN (Sabah):
		The supernatants were desalted and condensed in a dialysis bag with a 3500-cut-off molecular weight. The dialyzed protein was stored at −20°C until use.		LF: 2.80 ± 0.21 μg/mg; OVF: 1.31 ± 0.07 μg/mg;
				Cave red EBN (Sabah):
				LF: 3.43 ± 0.07 μg/mg; OVF: 3.13 ± 0.58 μg/mg;
				Cave red EBN (Sarawak):
				LF: 3.13 ± 0.07 μg/mg; OVF: 8.40 ± 0.56 μg/mg.
Haghani et al. (2016)	Teluk Intan, Perak, Malaysia.	The EBN extract were prepared based on Guo et al. (2006).	H-NMR spectra for flavonoid metabolites and sialic acid composition.	EBN from teluk Intan and Gua Madai have different derivatives of N-glucoloylneuraminic acid and N-acetyl-neuraminic groups.
	Gua Madai caves, Lahad Datu, Sabah, Malaysia.			Both EBNs have four sialic acid derivatives; N- glycol neuraminic acid, N-acetyl neuraminic acid, 5-N-acetyl-8, 9-di-O-acetylneuraminic acid and 5-Nacetyl-9-O-acetylneuraminic acid.
				EBN from Gua Madai consisted of Neu2,4,7,8,9 Ac6 (more O-acetylated branches).
				Only EBN from Gua Madai contained thymol-β-D glucopyranoside indicating antiviral activity against Herpes Simplex Virus and antibacterial property.
Zhao et al. (2016)	Nha trang, Khanh Hoa, Vietnam	**Grinding and Filtration**	Protein concentration by BCA Protein Assay.	The concentration of the extract’s protein was 0.75 mg/ml with a size of 31 KD.
		Nests were dried at 50°C for 24 h and grinded. The ground nests were kept in PBS at 30°C for 24 h. The suspension then was fully ground using a tissue grinder. Then it was centrifuged for 10 min at 3,000× *g*. The supernatant was collected and filtrate through to 0.2 µm filter.	Amino acid distribution (mg/g) was determined by L-8900 automatic amino acid analyzer.	
Haghani et al. (2017)	Teluk Intan, Perak, Malaysia	The EBN extract were prepared based on Guo et al. (2006).	Fluorometric HPLC	EBN from Gua Madai contained 6.7 mg/g of Neu5Ac.
	Gua Madai caves, Lahad Datu, Sabah, Malaysia			EBN from teluk Intan contained 3.2 mg/gr of Neu5Ac
Ghassem et al. (2017)	N/A	**Grinding**	DPPH, FRAP, ABTS and ORAC assay for antioxidant activity.	Various fractionated EBN samples (extract, crude hydrolysates, purified peptides) showed different antioxidant activities with purified peptide (<3 kDa) being the strongest antioxidant.
		Nest were pounded in a mortar and suspended in 1:5 (vol/vol) distilled deionized water and eluted for 24 h at 4°C.	LC-ESI-TOF MS/MS for peptide sequence identification.	Following peptide sequencing of <3 kDa fraction, two pentapeptides, PFHPY and LLGDP were discovered to have potent ORAC values. The peptide identified from the extract had amino acid sequences between 5 and 7 amino acids in length and the molecular weight between 514.29 and 954.52 Da. Most of them showed ORAC values > 5 μM of TE μM^−1^ peptide, indicating their antioxidative properties.
		**Heating**	MTT assay for *in vitro* cytotoxicity (cell viability with MRC-5 cell, oxidative rescue with HepG2 cell line).	PFHPY and LLGDP were not toxic to cells and showed protection against H_2_O_2_-induced oxidative damage in HepG2 cancer cells.
		The extract was boiled for and then proceed with enzymatic treatment.	Gastrointestinal proteases effects on antioxidant activity.	PFHPY and LLGDP maintained their potent antioxidant activity following exposure to gastrointestinal proteases.
		**Enzymatic treatment**		
		The EBN samples were treated with pepsin at 37 °C for 2 h with continuous shaking. The pH of the mixture was then adjusted to 8 and digested with trypsin for 2 h at 37 °C. The mixture was then boiled in water bath for 15 min.		
		**Centrifugation**		
		The mixture was centrifuged and the supernatants was collected, lyophilized as crude hydrolysate.		
		**Ultrafiltration**		
		The crude hydrolysate was then filtered using ultrafiltration cut-offs membranes producing 3 fractions with molecular weight of >30, 10–3, <3 kDa.		
		**Storage**		
		The samples were lyophilized stored at –80°C until further analysis.		
Noor et al. (2018)	EBN Grade A, B, C and D supplied by Mobile Harvesters Malaysia Sdn. Bhd	EBN of various grades were crushed and ground using home blender, then stored at room temperature.	Nutritional composition of moisture, protein, carbohydrate, ash and fat content.	Grade A EBN has highest protein and fat content (60.59 and 1.19%) followed by grade B, C and D. Grade C EBN has the highest carbohydrate content while Grade D EBN has the highest ash content.
	* A = Cleaned EBN, B = Semi-cleaned EBN, C = Washed Residue EBN, D = Unwashed EBN		Amino acid composition	All grade of EBN samples contained all essential amino acids in which valine, leucine, and threonine presented in high amount. Serine was the highest in four grades followed by aspartic acids. The least amino acids found in all EBN grades was methionine.
			Percentage recovery from hydrolysis	Grade A EBN demonstrated highest recovery of hydrolysate following hydrolysis (96–99%) followed by grade D, C and B.
Daud et al. (2019)	EBN supplied by Mobile Harvesters Malaysia Sdn. Bhd	N/A	Protein, carbohydrate and reducing sugar analysis	EBN samples have up to 60% of protein content, followed by carbohydrate (25%). About 6% of reducing sugar obtained from the total carbohydrate.
			FTIR spectroscopy for glycan	Glycan can be extracted following alkaline hydrolysis that remove significant protein amount.
			*In vitro* digestion	Consumption of glycan from EBN can resist stomach digestion in which it can pass into gut environment to act as prebiotic component.
Nuradji et al. (2018)	Kalimantan Island and Java Island, Indonesia	The EBN extract were prepared based on Guo et al. (2006) with some modifications on heating where aquabides (150 mg/ml) was used instead of distilled water.	Sialic acid content was analyzed by spectrophotometry.	The sialic acid content in EBN was 10.14% (w/w).
				EBN from Kalimantan island had higher sialic acid than EBN from Java island.
Babji et al. (2018)	Commercial EBN products from Pahang, Malaysia by Nest Excel Resources Sdn. Bhd.	**Heating**	Amino acid profiling.	Microbial analysis showed insignificant microbial activity probably due to gamma irradiation.
		EBN was soaked in a water with ratio 1:100 (w/v) and incubated for 16 h at 4°C, and followed by boiling for 30 min at 100°C and cooled to room temperature before pH adjustment.	Microbiological analysis and antioxidant and angiotensin converting enzyme (ACE) inhibitory assay.	Hydrolysates of EBN showed higher anti-oxidant ACE inhibitory activities.
		**Gamma irradiation**	LC-TSI-TOF mass spectrometry for identification of peptides.	Significant level of aromatic amino acid composition (histidine, phenylalanine, and tyrosine) and essential free amino acid such as valine and threonine in hydrolysate compared to the raw EBN.
		Irradiation of EBN powder using cobalt-60 irradiator (220 Gammacell^®^ Excel) at a rate of 2.17 kGy.h-1, at doses of 0.0, 1.0, 2.0, 5.0, 7.5, 10.0, 20.0 and 30.0 kGy.		Mass spectrometry identified aromatic amino acid (His, Phe, and tyr) composition, valine (Val), serine (Ser), proline (Pro), threonine (Thr) and phenylalanine (Phe).
		**Enzymatic hydrolysis**		
		1% enzyme alcalase was added to substrate, and followed by hydrolysis for 4 h and boiling of the hydrolysates for 5 min to inactivate the enzyme.		
		**Centrifugation**		
		The samples were centrifuged at 4,000 rpm, 4°C for 10 min.		
		**Filtration and storage**		
		The supernatants were filtered using Whatman No.1 and freeze-dried prior to storage for further analysis.		
		**Ultrafiltration**		
		The high and low molecular weight fractions of EBN protein hydrolysates were separated by ultrafilter membranes.		
Zulkifli et al. (2019)	Raw cleaned EBN obtained from terengganu supplied by Mobile Harvesters Malaysia Sdn. Bhd.	**Grinding**	Bradford assay to assess the soluble protein content.	Degree of hydrolysis was highest through addition of alcalase, especially at 0.5 and 1.5 h compared to others.
		Raw EBN was ground to powder and soaked in distilled water for 16 h at a ratio of 1:100 at 4°C.	DNS (3,5-dinitrosalyzed acid) method to determine reducing sugar in EBN hydrolysate.	Increase in hydrolysis time increased the solubility of EBN hydrolysate proteins, except for alcalase (1–3 h; not significant), and papaya juice (increase from 1 to 2 h, but decrease from 2 to 3 h).
		**Heating**	APPH assay for anti-oxidant activity.	Protein solubility of boiled EBN was lesser than hydrolyzed EBN.
		The samples were boiled through double boiling method at 100°C for 30 min, and cooled to room temperature before pH adjustment.	α-glucosidase inhibition to measure anti-hyperglycemic activity.	Hydrolyzed EBN contained higher reducing sugar concentration compared to boiled or unhydrolyzed EBN.
		**Enzymatic hydrolysis**		Increase in hydrolysis increased the anti-oxidant activity of EBN hydrolyzed by alcalase, however no such changes in EBN hydrolyzed with papain and papaya juice.
		1% alcalase (6 µL/100 ml), 1% papain (6 mg/100 ml) and 30% of papaya juice (30 g/100 ml) were added to EBN samples and was carried out for 0.5, 1.0, 1.5, 2.0, 2.5 and 3.0 h at 60°C. The enzymatic activities were stopped by boiling in water for 5 min.		EBN hydrolyzed with papaya juice showed moderate inhibitory activity against *α*-glucosidase activity whereas boiled and EBN hydrolyzed with alcalase and papain did not inhibit *α*-glucosidase activity.
		**Filtration and storage**		
		The samples were filtered using Whatman #4 filter paper, and frozen at -20°C for 48 h and then freeze-dried for further analysis. Boiled EBN without enzymatic treatment was freeze-dried and used as a control.		
Hun Lee et al. (2020)	Johor, Malaysia	**Heating**	Protein and amino acids analysi by SDS-PAGE and Waters AccQ Fluor Reagent kit (Waters).	EBN contained mostly of proteins (62–63%) and carbohydrates (25.62–27.26%).
		The feather of EBN samples were removed and dried in oven at 40 °C for 5 h to produce a constant weight.	HPLC-QTOF/MS for metabolites analysis.	Protein profile analysis showed 15 protein bands (16, 19, 21, 25, 27, 30, 37, 42, 49, 58, 66, 82, 97.4, 116 and 173 kD).
		**Grinding**	Fatty acids and triglyceride analysis by NMR spectroscopy.	EBN samples identified serine, aspartic acid, phenylalanine and tyrosine as the most abundant amino acids and the least amino acids of tryptophan and methionine.
		Dried EBN samples were blended and grounded into fine powder, then filtered through 1 mm steel filter.	DPPH, ABTS and catalase assay for antioxidantive, paraoxanase and anti-tyrosinase activities.	EBN samples were rich in poly-unsaturated fatty acids. The total amount unsaturated fatty acids accounted for 73.17% of overall fatty acids.
		**Storage**		EBN samples demonstrated strong free radical scavenging activity and paraoxonase activity with moderate anti-tyrosinase activity.
		Samples were stored at room temperature in air-tight container prior to analysis.		
Amiza et al. (2019)	Gong Badak, terengganu.	**Cleaning**	Degree of hydrolysis by optimizing temperature, pH, concentration, hydrolysis time.	The degree of hydrolysis (37.89%) was highest when EBN samples were enzymatically treated with 2% Alcalase, at 65°C, with a pH of 9.5 for 60 min.
		Raw uncleaned EBN was cleaned by soaking with water and rinsed frequently to remove contaminants and feathers.	Amino acid composition	Protein was the major nutritional component in dry raw and lyophilized EBN (70.55 and 67.63%). Ash and fat content were lower (except carbohydrate) in lyophilized EBN samples (hydrolysate) than raw EBN.
		Feathers were removed manually by using tweezers and the samples were kept in container and stored at -20 °C.		Sixteen types of amino acids were found in EBN hydrolysate. Valine was the highest essential amino acids in the samples while serine was for non-essential amino acids.
		**Heating**		
		Freeze EBN samples were homogenized with distilled water at 10,000 rpm for 2 min. Then, the samples were boiled for 30 min in water bath and let cooled.		
		**Enzymatic hydrolysis**		
		The homogenized EBN were treated with Alcalase using water bath shaker at a specified temperature and time. The treatment was inactivated at 85°C for 20 min.		
		**Storage**		
		The samples then were freeze-dried, and the resulting lyophilized samples were crushed into powder. The powder samples were kept in airtight container.		
Ling et al. (2020)	Commercial EBN products from Mobile Harvester (M) Sdn Bhd, Selangor, Malaysia	**Grinding**	Resorcinol method to determine N-acetylneuraminic acid level.	Recovery glycopeptides yield of clean EBN was 89.09 ± 0.01% and EBN co-products was 47.64 ± 0.26%.
		EBN clean and co-products were ground using a stainless-steel Waring blender for 2 min and kept in storage for further analysis.	Bradford protein assay to determine soluble protein content level.	The hydrolysate of clean EBN had the highest level of N-acetylneuraminic acid compared to the rest.
		**Enzymatic hydrolysis**	Fourier transform infra-red (FTIR) spectroscopy to identify functional groups in EBN.	Hydrolysate of EBN contained significantly higher level of soluble protein compared to RAW EBN, and FTIR spectra revealed carbohydrate and protein as the main components of EBN.
		3g of clean and EBN co-products were soaked in 100 ml distilled water and maintained for 16 h at 4 ± 1°C. This was followed by boiling at 90 ± 1°C for 30 min and cooled to 60 ± 1°C. Protease from Bacillus licheniformis was added to the solution and boiled for 5 min.	DPPH and FRAP assay to determine EBN’s antioxidant properties.	Clean EBN showed profoundly higher free radical scavenging activity (DPPH) and reducing power assay activity (FRAP) compared to EBN co-products.
		**Centrifugation**		
		The samples were centrifuged at 2610 X g for 10 min at 4 ± 1°C. Supernatants were freeze dried and their recovery were calculated.		
		**Storage**		
		The samples were stored in airtight container at room temperature.		
Gan et al. (2020)	Processed clean EBN supplied by Mobile Harvesters Malaysia Sdn. Bhd.	**Grinding**	Physicochemical analysis composed of water activity test, color analysis, viscosity test, degree of hydrolysis, s oluble protein content, and amino acid profiling.	The yield of EBN was highest in samples with freeze drying, followed by oven and spray drying.
		EBN were grounded using a cutting mill then passed through a 0.5 mm sieve.	Functional group identification *via* FTIR spectroscopy.	Oven drying demonstrated undesirable water activity, darker color and inferior bioactivities compared to spray and freeze drying.
		**Enzymatic hydrolysis**	DPPH, FRAP, ABTS assay for antioxidant activity.	Freeze drying method appeared to be the most desirable technique to achieve good EBN physicochemical properties and bioactivities.
		Grounded EBN samples were soaked in distilled water at room temperature. This was followed by boiling at 90°C for 30 min. Protease from Bacillus licheniformis was added to the solution and incubated in water bath.	Antihypertensive activity analysis.	Freeze-dried EBN samples showed the strongest free radical scavenging activity, ferric reducing antioxidant power but not ABTS scavenging activity.
		**Drying (oven, spray or freeze drying)**		No significant antihypertensive activity was found between different dried EBN.
		The samples were dried with either oven, spray or freeze drying.		
Hui Yan et al. (2021)	Clean EBN samples from Mobile Harvester (M) Sdn Bhd, Selangor, Malaysia	**Grinding**	Nutritional composition of EBN (protein, moisture, fat, ash and carbohydrate).	The majority of nutrional component of clean raw EBN consist of protein, followed by carbohydrate, moisture, fat and ash.
		EBN samples were crushed and homogenized to granules	Degree of hydrolysis and solubility	Increasing hydrolysis period will result in decreasing molecular weight of SiaMuc-glycoprotein (except at 60–90 min).
		**Double boiling**	SiaMuc-glycoprotein, protein and peptide content	Alcalase hydrolysis produce high recovery of bioactive SiaMuc-glycopeptide.
		EBN samples were made to swell by soaking in distilled water and stored overnight under 4°C. Then, the samples were double-boiled for 30 min.	Protein molecular weight profile	Alcalase hydrolysis produce high macro fractions of SiaMuc-glycoprotein thus increase the solubility and bioavailability of nutrients in EBN.
		**Enzymatic hydrolysis**		
		EBN samples were pre-heated in 60°C shaking water bath then, 1% alcalase was added while maintaining the pH level. Enzymatic treatment was investigated in four timepoints.		
		**Storage**		
		The samples were freeze-dried for storage.		

**FIGURE 1:**
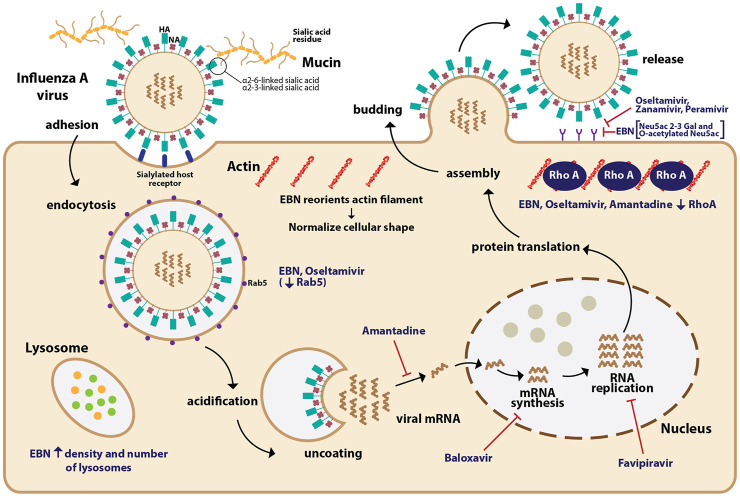
The mechanism of action of EBN and anti-viral drugs on influenza A virus (IAV). The IAV binds to the terminal sialic acid residues on mucins, particularly through α2-6 and α2-3 linkages leading to endocytosis of IAV into the host cell. The IAV core undergoes acidification allowing viral RNPs to be released into the cytoplasm. The IAV genetic material is transcribed and translated to produce viral materials, which once assembled, bud out from the infected cell. Anti-viral drugs such as baloxavir inhibit viral mRNA synthesis, favipravir inhibits RNA replication, amantadine inhibits the release of viral mRNA, and neuraminidase inhibitors (oseltamivir, zanamivir, and peramivir) inhibit the release of the IAV. EBN increases the density and number of lysosomes in infected cells. Similar to oseltamivir, EBN reduces the expression of Rab5, which facilitates the endocytosis of IAV. EBN reorients the actin filament and normalizes the cellular shape of infected cells. Similar to oseltamivir and amantadine, EBN also reduces the expression of RhoA, a protein involved in actin filament polymerization. EBN, through its sialic linkages such as Neu5Ac 2-3 Gal, and O-acetylated Neu5Ac may prevent the release of IAV.

For its anti-viral effect, EBN water extracts significantly reduced hemagglutination activity of IAV (H1N1, H3N2, and H5N1) in a dose-dependent manner (Guo et al., 2006; Haghani et al., 2017; Nuradji et al., 2018), with increasing efficacy when the treatment duration is prolonged (Haghani et al., 2017). The efficacy of EBN with enzymatic treatment (pancreatin F), and without enzymatic treatment was the focus of many *in vitro* investigations. EBN without enzymatic treatment reduced extracellular NA copy number, NS1 expression, and at the same time also increased intracellular NS1 expression. EBN with enzymatic treatment increased intracellular NA copy number, increased extracellular NS1 expression, and increased intracellular M2 expression, suggesting the effects of EBN with enzymatic treatment (Pancreatic F) are more inclined to inhibiting the release of the viral materials (Haghani et al., 2016). EBN (from house nest) with and without enzymatic treatment reduced virus titer, with a percentage of protection 42.47 ± 8 and 45.42 ± 8.4, respectively (Haghani et al., 2017). In line with these findings, Guo et al. (2006) reported significant inhibitory effects of EBN with pancreatic enzyme digestion on hemagglutination activity of IAV, whereas little effects when treated with EBN without the pancreatic enzymes. The findings indicate that EBN extracts with smaller proteins (10–25 kDa) when treated with the pancreatic enzyme produced stronger inhibitory effects compared to the larger proteins (more than 50 kDa) expressed in the EBN without enzymatic treatment. The same researchers also reported that EBN extracts (4 mg/ml) did not inhibit the neuraminidase activity of influenza, and therefore, the EBN extracts act on viral hemagglutinin but not viral neuraminidase (Guo et al., 2006). In addition to reducing viral load, EBN also normalized the cellular shapes and reoriented the actin filaments, and reduced the densities of actin filaments caused by the IAV infection. EBN also increased the density and the number of lysosomes in both infected and non-infected cells (Haghani et al., 2017) (Figure 1).

Treatment with another enzyme, neuraminidase significantly affected the inhibitory effects of EBN (Guo et al., 2006), suggesting the important role of glycosidic linkages in the anti-viral properties of EBN. Lectin blotting assay revealed Neu5Ac2-3Gal linkage as the major molecular species of sialic acid in the EBN extract along with O-acetylated Neu5Ac (Guo et al., 2006). Based on the location of collection (house vs cave), EBN extracts differ chemically, where EBN collected from the cave (Gua Madai) was reported to contain Neu2, 4,7,8,9 Ac6, which has more O-acetylated branches (Haghani et al., 2016). O-acetylation of N-acetyl residues confers higher resistance of sialic acids to the actions of sialidases from various infectious agents (Corfield et al., 1981; Haverkamp et al., 1982). EBN from Gua Madai recorded the highest percentage of protection against IAV compared to EBN collected from House Nest. Fluorometric High-Performance Liquid Chromatography revealed EBN from Gua Madai contained 6.7 mg/g of Neu5Ac, whereas EBN from House Nest contained 3.2 mg/g of Neu5Ac (Haghani et al., 2017). Both research groups suggest that the higher amount of sialic acid and the diversity of its derivatives increase the anti-viral effects of EBN.

## Cytokine Storm in Influenza and COVID-19 Infections

Cytokine storm (CS) or aberrant pro-inflammatory responses is a known complication in severe influenza (Woo et al., 2010; Ishikawa, 2012; Kalaiyarasu et al., 2016) and COVID-19 cases (Chan et al., 2020; Wang Z. et al., 2020; Zhao et al., 2020). Infected cells undergoing apoptosis and necrosis trigger acute inflammatory response recruiting leukocytes and plasma cells to the site of infection (Knipe and Howley, 2013). Hence, increasing the production of pro-inflammatory cytokines and chemokines (La Gruta et al., 2007; Shinya et al., 2012) *via* transcription factors such as Nf-κB, AP-1, interferon response factors 2 and 7 (Thompson et al., 2011).

In severe influenza cases, increase in transcription activities of NF-κB and IRF 3/7 promote anti-viral pro-inflammatory responses in the lungs, including excessive levels of interferon, tumor necrosis factor (TNF-α), interleukin (Beigel et al., 2005) (IL-1 and IL-6) (Tisoncik et al., 2012), IL-8 (de Jong et al., 2006), and chemokines such as MCP-1, RANTES, causing severe influenza-induced immunopathy (Berri et al., 2014; Chiang et al., 2014) Increased activities of NF-κB also escalate the expression of IκB (an inhibitor of NF-κB) and anti-inflammatory cytokines to attenuate the NF-κB-induced pro-inflammatory responses. Early responses to IAV infections indicate a rise in the levels of RANTES, MCP-1, IL-8, IFN-α, IFN-β, and IFN-κ (Le Goffic et al., 2007; Xagorari and Chlichlia, 2008), whereas late responses have recorded a surge in IL-1α/β, IL-6, TNF-α, IL18, IFN type I, MCP-1, MIP-1α, MIP-1β, RANTES, MCP-3, MIP-3α, and IP-10, especially secreted by macrophages residing in the lower respiratory tract (Jewell et al., 2010). Such changes in cytokine waves tip the balance between pro-inflammatory and anti-inflammatory responses, along with viral virulence causes respiratory epithelial injuries such as acute lung injury or even ARDS (Shimizu, 2019) reducing oxygen saturation level leading to mortality (For review: Gu et al., 2019).

In COVID-19 cases, an increase in IL-6 level was most frequently associated with the severity of the disease (Chen C. et al., 2020; Gao et al., 2020; Ruan et al., 2020) and other cytokines and chemokines, including IL-1B, IL-7, IL-8, IL-9, IL-10, FGF, G-CSF, GM-CSF, IFN-γ, IP-10, MCP-1, MIP-1A, MIP1-β, PDGF, TNF-α, and VEGF (Huang et al., 2020) were also documented. At the early stage of SARS-CoV infection, there is a delay in the release of chemokines, cytokines, hence a low level of IFN. At the same time, the abundant release of pro-inflammatory cytokines such as TNF, IL-1β, IL-6, and chemokines such as CCL-2, CCL03, CCL-5 causes excessive infiltration of inflammatory cells into the lungs (Law et al., 2005; Lau et al., 2013). Pre-clinical findings revealed an early delay in the release of IFN-α/β induced generation of more monocyte chemoattractants such as CCL-2, CCL-7, and CCL-12 by macrophages causing their further accumulation, and production of pro-inflammatory cytokines, including IL-1β, IL-6, TNF-α, and inducible nitric oxide synthase. Cumulatively, IFN-α/β and IFN-γ cause apoptosis of T cells (Channappanavar et al., 2016), airway epithelial cells, alveolar epithelial cells, and endothelial cells (Shimizu, 2019) damaging pulmonary vasculature, leading to vascular leakage and alveolar edema, causing hypoxia (Channappanavar et al., 2016; For review: Ye Q. et al., 2020). Endothelial injuries also cause spillover of cytokines and chemokines into the blood circulation causing multi-organ failure (Tisoncik et al., 2012). In line with this, findings from non-human primates revealed that SARS-CoV-infection-related death is more likely due to excessive immune response rather than virus titer (Smits et al., 2010).

## The use of Corticosteroids in Influenza and COVID-19 Infections

The severity of- and mortality due to influenza and COVID-19 infections are determined by the host resistance and virulent factors, where severe infections usually trigger hyperactive host resistance, hence causing adverse symptoms (Liu et al., 2020), such as acute respiratory distress syndrome (ARDS), eventually leading to mortality. Influenza patients with ARDS are managed through corticosteroid therapy for their anti-inflammatory actions. Over the years, a myriad of systematic and meta-analyses have consistently reported that routine corticosteroid treatment in influenza patients may not be appropriate. Many reported a significant association between corticosteroid treatment and higher mortality (Zhang et al., 2015; Ni et al., 2019; Lansbury et al., 2020; Zhou et al., 2020), whereas some have reported that low-to-moderate dose of corticosteroids may reduce mortality (Li et al., 2017). Some have claimed that early and high-dose corticosteroid use is associated with hospital mortality (Lansbury et al., 2020; Sheu et al., 2020; Tsai et al., 2020). In addition to high mortality, corticosteroid treatment also correlated to secondary infections (Yang et al., 2015; Lansbury et al., 2019; Ni et al., 2019; Zhou et al., 2020) due to prolonged hospital stay (Yang et al., 2015; Ni et al., 2019).

Systematic review and meta-analyses of randomized clinical trials revealed that corticosteroids reduced the mortality in critically ill COVID-19 patients (WHO Rapid Evidence Appraisal for COVID-19 Therapies (REACT) Working Group et al., 2020; Ma et al., 2021; van Paassen et al., 2020), especially patients with compromised lung capacity (Bartoletti et al., 2021), however with delayed viral clearance and increased secondary infections (van Paassen et al., 2020). Some have also reported increased short-term mortality following corticosteroid use in COVID-19 patients with ARDS (Liu et al., 2020), whereas some found no significant association between corticosteroid use and mortality (Shuto et al., 2020; Albani et al., 2021). In line with these, some have warned against the use of corticosteroids in non-critically ill COVID-19 patients due to longer hospitalization, longer duration of viral shedding, and progression of non-severe to severe cases (Shuto et al., 2020; Yuan et al., 2020). Due to many limitations such as study designs, sample size, age factors, viral strain, and pharmacogenomics discrepancy, at present, it is not empirically possible to conclude the efficacy of corticosteroid use in COVID-19 or influenza patients.

## Anti-Inflammatory Effects of EBN

**FIGURE 2:**
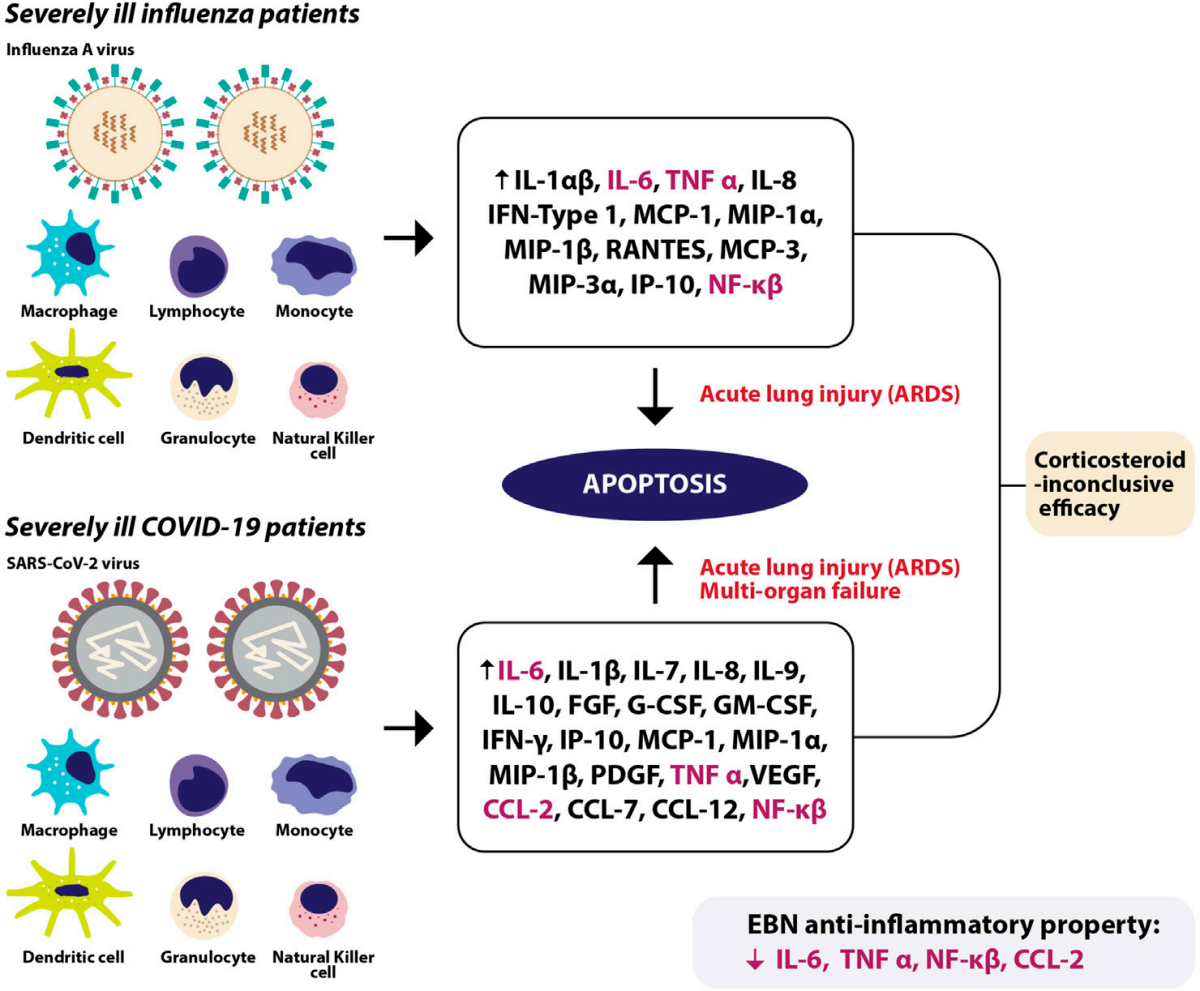
In severely ill influenza patients, immune cells release various cytokines and chemokines leading to acute lung injury, or also known as acute respiratory distress syndrome (ARDS) due to apoptosis. In severely ill COVID-19 patients, various immune cells release a myriad of chemokines and cytokines causing apoptosis, leading to ARDS and spillover of cytokines and chemokines into the blood circulation causing multi-organ failure. Among the escalated cytokines and chemokines in severe influenza and COVID-19 infections, EBN was shown to reduce IL-6, TNF-α, CCL-2, and NF-κβ at the pre-clinical level.

EBN (House nest from Teluk Intan), when tested for its anti-viral effects using an *in vivo* model, significantly increased the expression of IFN-γ, IL-1β, IL-2, IL-6, and TNF-α on day 1 following infection, significantly decreased the level of IFN-γ, and upregulated IL-4, IL-6, IL-12, and TNF-α on day 3 post-infection, and reduced IL-10 and IL-12 on day 7. The profound increase in the pro-inflammatory cytokines seen during the early stage of infection is similar to numerous anti-viral medications, including the anti-viral drug oseltamivir used in the same study. On post-infection day 5 and 6, the level of IL-6 remained high in the oseltamivir treated groups, unlike the EBN treated groups where the levels of pro-inflammatory cytokines were well regulated. In the same study, EBN (House nest from Teluk Intan) treated with pancreatic F enzyme significantly decreased the levels of IL-6 and chemokine (C-C motif) ligand 2 (CCL2) *in vitro* (Haghani et al., 2016). In line with these findings, studies using other disease models also reported EBN-induced inhibition of nitric oxide and TNF-α production in LPS-stimulated macrophages (Aswir and Wan Nazaimoon, 2011; Vimala et al., 2012), improvement in the proliferation of lymphocytes and splenocytes (Zhao et al., 2016), free radical scavenging activities and reduced pro-inflammatory markers such as CRP, TNF-α, CCL2, NF-κB (Yida et al., 2015) (Figure 2). Table 5 shows the anti-inflammatory effects of EBN on various models.

**TABLE 5:** Anti-inflammatory effects of EBN.

References	Cell lines	Origin of EBN and dosage	Experimental design	Analysis	Summary of findings
Aswir and Wan Nazaimoon (2011)	Human colonic adenocarcinoma cell line (Caco-2 cells) and mouse leukemic monocyte macrophage cell line (RAW 264.7)	Two processed, commercial brands (Y1 and X1) and 4 unprocessed samples obtained from 3 zones [North (Zua1 and Zub1), South (ZS1) and East Coast (ZP1)] of Peninsular Malaysia.	EBN treatment was grouped based the brand and origin of the samples, and the working concentration of the EBN used was 5 ppm.	Proliferative effect of EBN was assessed using Caco-3 cells.	Significant cell proliferation (*p* < 0.05) was noticed in cells treated with 5ppm of unprocessed or commercial EBN. The highest proliferation was seen in treatment with EBN (Brand X1) at 215.07 ± 4.74%.
				Anti-inflammatory effect of EBN was assessed using RAW 264.7 cells.	Sialic acid (0, 2, 4, 6, 8 and 10) caused a dose dependently increased the proliferation of Caco-3 cells. At 24h, 2% of sialic acid significantly increased cell proliferation by 50% (*p* = 0.027), and 10% of sialic acid increased proliferation by more than 100% (*p* = 0.009).
					Brand Y1 reduced the percentage of TNF-α production to 43% (430 pg/ml), EBN from East Zone and South Zone reduced TNF-α production to 31% (310 pg/ml) and 24% (240 pg/ml), respectively. GlcNA was the most effective in reducing TNF-α production at 2ppm.
Vimala et al. (2012)	Mouse macrophage cell line RAW 264.7.	Two processed brands of EBN (Brand X and Y) and eight unprocessed raw (ECZ1, ECZ2, NZ1, NZ2, NZ3, SZ1, SZ2 and SZ3) white EBN from three zones in Peninsular Malaysia; two from East Coast, three from North and South zones	The treatment groups were categorized based on the types of EBN.	Nitrite (NO) production	All EBN extracts inhibited TNF-⍺ and NO production in LPS-stimulated RAW 264.7 macrophages. ECZ1 and ECZ3 showed cell viability over 80% by the MTS assay.
			RAW cells were then treated with EBN at 1, 10 and 100 ppm for 2 h and cultured with LPS from Escherichia coli serotype 0111: B4 (0.1 mg/ml) for 24 h at 37°C.	TNF-⍺ concentration.	
				Cell viability by MTS assay.	
Yew et al. (2014)	SH-SY5Y cells.	Crude and water extract of EBN from Perak, Malaysia.	S1 (crude) and S2 (Water extract) of EBN used throughout the study.	Cell viability by MTT assay.	Pre-treatment with EBN extracts and co-incubation with 6-OHDA generally did not significantly improve the viability of cells. Pre-treatment with S1 or S2 prior to 6-OHDA protected SH-SY5Y cells by decreasing cell death, nuclear apoptotic changes in the culture.
			SH-SY5Y cells were exposed to 6-ODHA to induce cytotoxicity and treated with S1 and S2 EBNs.	Morphological examination.	High dose S2 treatment instead of S1 significantly reduced ROS level. The level of ROS was maintained at baseline when SH-SY5Y cells were treated with EBN extracts alone.
				Intracellular ROS level.	S1 increased apoptotic event in untreated cells and did not reverse the 6-OHDA induced early apoptosis. However, S2 reduced the early apoptosis events and attenuated 6-OHDA induced caspase-3 activation. No significant difference in MMPs between the EBN and 6-OHDA groups.
				Apoptosis analysis, mitochondrial membrane potential assessment, caspase-3 detection.	
Yida et al. (2015)	Sprague dawley rats (10-week-old, 230–280 g).	EBN from terengganu, Malaysia.	The rats were fed high fat-diet (HFD)containing	Thiobarbituric acid reactive species (TBARS).	Both EBN treatments significantly improved percentage of scavenging activity and reduced liver MDA level compared to HFD group.
			4.5% cholesterol and 0.5% cholic acid with or without treatment using Simvastatin or EBN (2.5, 20%) for 12 weeks, except the normal group.	Serum total antioxidant.	Both EBN treatments significantly reduced HFD-induced increase in CRP, IL-6 and TNF-⍺ and reduced serum inflammatory markers CRP and TNF-α but had little effects on serum total antioxidant status.
				Serum C-reactive protein (CRP), IL-6 and TNF-⍺.	EBN increased hepatic antioxidant genes suppressed by HFD, such as superoxide dismutase [SOD] 1 and 2, glutathione reductase (GSR), and glutathione peroxidase
				Gene expression study.	(GPx), and reduced HFD-induced increase in hepatic inflammatory genes CRP, TNF-α, CCL2, and NFκβ.
Hou et al. (2015b)	SH-SY5Y human neuroblastoma cell line.	EBN from Sabah and Sarawak, Malaysia.	SH-SY5Y cells were treated with varying doses (depends on the assay) of EBN, lactoferrin (LF) and ovotransferrin (OF), respectively.	ABTS radical cation scavenging activity.	EBN (1000 μg/ml) produced the greatest scavenging effect on ABTS followed by LF and OF.
				Oxygen Radical Absorbance Capacity (ORAC) Assay.	EBN showed more than 80% viability on SH-SY5Y cells with 1000 μg/ml EBN producing the greatest effects.
				MTT assay.	Cell appeared normal in EBN, LF, and OF treated cells while H_2_O_2_ caused early apoptosis.
				Acridine orange and propidium iodide (AO/PI) staining (Cell apoptosis).	Pretreatment with LF, OVF, and EBN for 24 h recovered the SOD activity compared to H_2_O_2_ treated cells where SOD decreased. EBN reduced intracellular H_2_O_2_.
				Superoxide dismutase (SOD) and ROS ELISA assays.	EBN upregulated the expression of SOD1, SOD2, and PARP1 genes even in the presence of H_2_O_2_.
				Real-time Polymerase Chain Reaction.	No significant differences in SOD mRNA levels between EBN, LF, and OVF.
Zhao et al. (2016)	BALB/c mice (female, 18–22 g, 6–8 weeks old).	EBN from Nha trang, Khanh Hoa, Vietnam.	Treatment groups:	*In vitro*	All doses of EBN significantly increased the proliferation of lymphocytes and splenocytes. All EBN extracts improved proliferation of splenocytes.
			*In vitro*	Cell proliferation	Different EBN extracts concentrations markedly elevated percentages of CD19+/CD25+, CD19+/CD71+, CD19+/CD69 + cells compared to control.
			Splenocyte and B lymphocytes collected from BALB/c mice and cultured.	Immunoglobulins detection	EBN extracts promote activation of B-cells in the early, middle, and later periods.
			*In vivo*	*In vivo*	EBN elevated IgA, IgE, IgG3, and IgM levels, but not IgG1, IgG2a, and IgG2b compared to the control group.
			Control, cyclophosphamide (CY)-treated animals on Day 27.	IgA concentration in intestinal lavage and cD3+/cD19 + lymphocytes among Peyer’s patch cells.	All EBN doses significantly increased intestinal secretion of sIgA level compared to CY-treated group, with mid dose showing the greatest effects.
			EBN (low dose (EBNL, 0.42 g/kg), medium dose (EBNM, 0.83 g/kg), and high dose (EBNH, 1.66 g/kg).		All doses of EBNE significantly reversed the CY-induced drop in CD3^+^ T- and CD-19+ B-cells in Peyer’s Patch cells with mid dose showing the greatest effects.
			EBN was given for 28 days, and on day 27 the mice were given CY to induce immunosuppression followed by removal of their small intestine after 24 h.		
Ghassem et al. (2017)	HepG2 (human hepatocarcinoma cell line)		Different cell lines were treated varying concentration of purified peptides from EBN extract.	Antioxidant assay.	EBN showed effective radical-scavenging and promising antioxidant properties.
	MRC-5 (human embryonic fibroblast cell line)			Cytotoxicity assay.	Two novel pentapeptides Pro-Phe-His-Pro-tyr and Leu-Leu-Gly-Asp-Pro, corresponding to f134–138 and f164–168 of cytochrome b of *A. fuciphagus* indicated the highest ORAC values indicating increased scavenging activity.
				ORAC assay.	Scavenging activity increased following hydrolysis (digestive enzymes) and ultra-fractionation due to more cleavage and increased number of low molecular weight peptides. The antioxidant potency of PFHPY and LLGDP peptides was not affected by *in vitro* incubation with gastrointestinal enzymes.
					No cytotoxicity effect of purified peptides at concentration was observed. Pretreatment with purified peptides enhanced the HepG2 cell viability.

The discrepancy in the therapeutic potential of EBN of various sources and preparation techniques should be taken into consideration. For an instance, some researchers have reported increased viability of cells following incubation with EBN (Zhao et al., 2016; Haghani et al., 2017) with increasing concentration of sialic acid (Aswir and Wan Nazaimoon, 2011). Whereas, some reported no improvement in viability of cells following co-incubation of EBN (water extract) with 6-hydroxydopamine (Yew et al., 2014). However, when incubated with crude EBN, 20% of viability was seen (Yew et al., 2014). Moreover, treatment with high-dose crude EBN significantly promoted intracellular production of ROS (twice than 6OH treated level). However, high-dose water extracts of EBN significantly reduced the ROS level (Yew et al., 2014). In mice, all EBN dosages employed in a study induced six division cycles of CD-19 stained (B cells). EBN (0.19, 0.38, and 0.75 mg/ml) only completed one division of CD-3 stained (T cells) cells, indicating that the immunomodulatory property of EBN could be more directly inclined to B cell responses (Zhao et al., 2016).

## Bioactive Ingredients in EBN

EBN mainly consists of protein (almost 60%), carbohydrate (25%) (Daud et al., 2019), moisture, fat, and ash (Amiza et al., 2019; Hun Lee et al., 2020; Hui Yan et al., 2021). At present, the potential bioactive ingredients in EBN with anti-viral and immunomodulatory properties are glycoprotein or glycopeptides. Sialylated N-glycan was identified as the main component of EBN contributing to its anti-viral property (Yagi et al., 2008). Fluorometric analysis revealed Neu5Ac 2-3 Gal linkages in the cave and O-acetylated Neu5Ac in house-cultured EBN (Guo et al., 2006). Further analysis identified derivatives of sialic acid such as N-glycol neuraminic acid, N-acetyl neuraminic acid, 5-N-acetyl-8,9-O-acetylneuraminic acid, and others such as thymol-β-glycopyranoside in EBN (Haghani et al., 2016). The importance of sialic acid in human nutrition is well known (Ghosh, 2020). Hydrolysis of EBN produced the highest amount of soluble protein compared to other forms of EBN. The hydrolysates of EBN contained high level of N-acetylneuraminic acid with profound anti-oxidant effects (Gan et al., 2020). The degree of hydrolysis was the highest when EBN was enzymatically treated with alcalase (Amiza et al., 2019; Zulkifli et al., 2019; Hui Yan et al., 2021), recovering a high level of bioactive Siamuc-glycopeptide (Hui Yan et al., 2021), and amino acids including valine, serine, aspartic acid, phenylalanine, tyrosine, histidine, threonine, and leucine (Babji et al., 2018; Noor et al., 2018; Amiza et al., 2019; Hun Lee et al., 2020). In line with this, the anti-oxidant activity of enzymatically treated EBN was significantly higher, especially alcalase compared to just boiled EBN (Babji et al., 2018; Zulkifli et al., 2019). Other glycoprotein and protein such as ovotransferrin, and lactoferrin also identified in EBN, especially higher in house and cave white EBN compared to cave red and black EBN (Hou et al., 2015a). Ovotransferrin is known for its anti-bacterial, anti-viral, and anti-inflammatory properties (Giansanti et al., 2016). Similarly, lactoferrin from milk also been well explored for its anti-viral and anti-bacterial activities (Giansanti et al., 2015).

Most of the studies investigating the potential medicinal values of EBN used water extract (Haghani et al., 2016; Haghani et al., 2017), as bioactive compounds in EBN are water-soluble. Water extraction of EBN results in a mixture of glycopeptides (Guo et al., 2006; Yagi et al., 2008; Ling et al., 2020; Hui Yan et al., 2021) which should be isolated or purified through a binding affinity technique utilizing specifically targeted ligands anchored to the solid phase such as a polyester membrane or cellulose-based surface. The purified or isolated bioactive ingredients can then be released by elution buffer with an appropriate pH. In a large-scale setting, it would be more practical to carry out molecular size range separation (Babji et al., 2018) prior to the purification or isolation process, to reduce the complexity of the extract and slow down the purification step. By fractioning the EBN extract based on their molecular size, the purification step can be further simplified. This should be followed by the identification of bioactive compounds in the fractions. House harvesting of EBN results in various grades of EBN (Grade A-D) (Noor et al., 2018). The fragments and crushed pieces of EBN are of low value for conventional EBN processing. Nonetheless, this EBN might be more applicable as raw material for EBN extract preparation, which involves grinding the EBN into powder, and followed by soaking in water to increase their surface area for reaction with water or enzymes. Subsequently, the crude extract should be subjected to fragmentation based on molecular size and followed by purification. Purified peptides (3 < kDa) showed the strongest anti-oxidant activity compared to extract and crude hydrolysate (Ghassem et al., 2017). Techniques such as freeze-drying removes water while preserving the functional groups of the bioactive compounds. In line with this, freeze-drying has been reported to result in a high yield of EBN compared to oven and spray drying (Gan et al., 2020). The glycopeptides in EBN are easily denatured by the heat drying method, hence the water content should be evaporated under low temperature to preserve the bioactive compounds. Upon usage, the compounds can be reconstituted easily with water to regain their functional property.

## EBN Studies in Humans: What we Know so far

EBN is an expensive delicacy for the Chinese and sometimes is considered as the “Caviar of the East” (Marcone, 2005). EBN is exclusively popular as part of the traditional Chinese medicine to treat respiratory ailments, moistening the lungs, heart tonics, and stomach nourishments (Zhao et al., 2016). Owing to its documented effects in the old Chinese medical textbooks, the Chinese frequently consumed EBN as a supplement for health benefits (Zhao et al., 2016). Numerous authentic EBN products, particularly in a form of extract or essence, were sold over the counter where most of them were imported exclusively from the Southeast Asian regions (Dai et al., 2020). To date, no findings have directly related the health benefits of EBN in humans. The closest to human testing were the *in vitro* setups involving cultured human cell line such as keratinocytes (Kim et al., 2012; Hwang et al., 2020), SH-SY5Y cells (Hou et al., 2015b), lung carcinoma cells (Guo et al., 2006), colonic adenocarcinoma cells (Aswir and Wan Nazaimoon, 2011), chondrocytes (Chua et al., 2013), and hepatocarcinoma cells (Ghassem et al., 2017). *In vivo* reports were derived from pre-clinical animal models (Yida et al., 2015; Zhao et al., 2016). Hence, the potential benefits of EBN in humans are yet to be proven empirically. In line with human consumption, EBN was reported to cause allergic reactions in pediatric patients due to avian allergen homologous to ovoinhibitor precursor in chicken (66-kd protein) and potentially by other additives or contaminants (Goh et al., 2000; Goh et al., 2001). Compared to fresh EBN extract, commercially prepared EBN showed an undetectable amount of allergen in immunoblot probably due to boiling of the EBN, however still able to trigger an allergic reaction, indicating that the allergen is not heat sensitive. Periodate treatment relinquished the Ig-E binding ability of the allergen suggesting that allergenic epitope could be due to carbohydrate moiety which is known to be resistant to cooking and boiling (Goh et al., 2001). Contaminants such as fungus also been reported to present in EBN, especially plant and soil fungi in raw EBNs and environmental fungi in commercial and boiled EBNs (Chen et al., 2015).

## EBN as a Potential Anti-Viral and Anti-Inflammatory Therapies: Future Direction

The anti-viral efficacy of EBN was compared across standard anti-viral medications such as oseltamivir and amantadine, and similar to those drugs EBN also reduced protein involved in viral trafficking such as Rab5 (not amantadine) and protein involved in actin filament polymerization, RhoA. EBN also inhibited the HA activity of the virus (Guo et al., 2006; Haghani et al., 2017; Nuradji et al., 2018). The anti-viral drugs currently being used to treat IAV infection are baloxavir marboxil (inhibits viral mRNA synthesis) (Shionogi and Co. Ltd, 2018), oseltamivir, zanamivir, and peramivir (selective neuraminidase inhibitors) (Gubareva et al., 2000; Ohuchi et al., 2006; Hama, 2015), and favipravir (inhibits RNA polymerase activity) (Furuta et al., 2013). Apart from the neuraminidase inhibitors, it is yet to be explored if a combined therapy of EBN and other classes of anti-viral medications could achieve a synergistic outcome. EBN also attenuated the surge in pro-inflammatory cytokines and chemokines such as TNF-α, CCL-2, NF-κβ, NO, IL-6, and increased IFN-γ (Aswir and Wan Nazaimoon, 2011; Vimala et al., 2012; Haghani et al., 2016), which are dysregulated in severe forms of IAV (Xagorari and Chlichlia, 2008; Tisoncik et al., 2012), and SARS-CoV infections (Gao et al., 2020; Huang et al., 2020). Moreover, EBN also ameliorated apoptosis (Yew et al., 2014), and normalized the cellular shape of IAV-infected cells (Haghani et al., 2017), which may reduce the collateral damage to the host cells in severe infections. Furthermore, EBN also exhibited anti-bacterial property (Hun et al., 2015), and improved B cell activity (Zhao et al., 2016), which may provide additional protection against opportunistic bacterial infections in cases of immunosuppressive use in severely ill patients. Nonetheless, to date, the efficacy of EBN as anti-inflammatory agent is yet to be compared empirically with clinically used anti-inflammatory medications. The allergenic effect of EBN also should be taken into account (Goh et al., 2001), considering the already known high risk of cytokine surge in severe IAV and COVID-19 patients. Techniques such as ultrafiltration tend to isolate larger glycoproteins, with higher allergenic potential, and periodate treatment relinquished the allergenic activity of EBN (Goh et al., 2001). It is imperative that future investigations should further explore how these techniques affect the bioactive yield of EBN, and their health-promoting effects in living organisms. The presence of contaminants such as fungus (Chen et al., 2015), and residual contaminants (Yeo et al., 2021) should be reflected in pre-clinical settings prior to clinical application. Gamma irradiation reduced the microbial activity of EBN to an undetectable level (Babji et al., 2018), whether this equally affects the efficacy of EBN in various *in vivo* disease models should be investigated further.

## Conclusion

The anti-viral and anti-inflammatory property of EBN is promising at the pre-clinical level. Nevertheless, future studies should consider the EBN’s potential allergenic and contaminant factors in their study designs prior to its clinical application.
